# The effects of empathy by caregivers on healthcare service satisfaction

**DOI:** 10.3389/fpsyg.2022.912076

**Published:** 2022-10-06

**Authors:** Xiaoyi Wang, Ruining Wang, Feng Sheng, Leyi Chen

**Affiliations:** ^1^School of Management, Zhejiang University, Hangzhou, China; ^2^Faculty of Arts, McGill University, Montreal, QC, Canada

**Keywords:** service satisfaction, physician–patient communication, empathy, patient-centered care, patient autonomy, electroencephalography (EEG)

## Abstract

Healthcare service satisfaction focuses not only on the patients but also on the caregivers’ perspectives. This study explored how caregivers’ empathy toward patients affects their satisfaction with healthcare services through an electroencephalography (EEG) experiment. EEG mu rhythm was used as the neural indicator to reflect empathy. The results showed that empathy reduces caregivers’ evaluation of healthcare service satisfaction because they share suffering with the patients. However, implementing physician–patient communication through a process-based informed consent (IC), compared to an event-based IC, can effectively alleviate such adverse effects.

## Introduction

As one of the largest and fastest-growing industries in the world, the healthcare services market size is expected to grow from $6.87 trillion in 2021 to $7.55 trillion in 2022 at a compound annual growth rate (CAGR) of 9.8% and reach $10.41 trillion in 2026 at a CAGR of 8.4% (Healthcare Services Market Analysis, Size And Trends Global Forecast 2022-2026, 2022). Most patients have a caregiver (parents, spouses, or other family members) during their medical visits, especially children and elderly patients (Clayman and Morris, 2013; Turabian et al., 2016; Riffin et al., 2021). Healthcare service satisfaction has traditionally focused on patients’ views, while caregivers’ perspectives are little studied. In the patient–physician relationship, caregivers often play essential roles in providing emotional, informational, or practical support for the diagnosis and treatment decisions (Laidsaar-Powell et al., 2013). Therefore, it is essential to assess the satisfaction from the caregivers’ perspectives to improve the quality of healthcare service.

In addition to the physician’s medical ability, caregivers’ satisfaction with healthcare service also depends on their ability to empathize with the patients. Empathy enables caregivers to experience the patients’ affective state, engage in their cognitive process, or even behave empathically (Zaki and Ochsner, 2012; Clark et al., 2019), which, in turn, influences the evaluation of the clinical service. This study aims to explore the impact of empathy toward patients on caregivers’ satisfaction with the healthcare service. Notably, empathy can be subjected to various modulations (Hein and Singer, 2008; Szanto and Krueger, 2019). For example, the strength of the empathy response could be modulated by features of emotions (valence, intensity, and saliency), the relationship between empathizer and target (affective link and nurturance, familiarity and similarity, and communicative intentions), situation context (appraisal of the situation and display of multiple emotions), empathizer (mood arousal, emotional regulation capacities, personality, gender, and age), etc. (de Vignemont and Singer, 2006; Yalçın and DiPaola, 2020). To investigate the impact of empathy more nuancedly, we identified how to modulate empathy. In a healthcare service setting, we specifically compared empathy under two important modulators: physician–patient communication style and disease severity.

With self-reported data, one limits their understanding to, for example, attitudes or perceptions. Recently, several calls have been launched to fuse service research with neuroscientific insights (Van Vaerenbergh et al., 2019). Non-invasive brain scan methods can enhance our understanding of how humans experience various service elements, such as sounds, communication, and visual aspects (Bolton et al., 2018). In different neurological measurement tools, electroencephalography (EEG) can accurately detect internal processes and mechanisms. Moreover, self-reported data cannot assess psychological responses in real time, while EEG can reveal information at a millisecond temporal resolution (Spiegelhalder et al., 2014; Plassmann et al., 2015). Some studies have used EEG, which measures voltage fluctuation at the brain’s surface for empathy recognition (Cheng et al., 2014; Fabi and Leuthold, 2017; Peled-Avron et al., 2018). Combining self-reported data with neuroscientific signals can increase the validity and understanding of one’s mental state. To this end, we performed an EEG experiment in this research and measured some self-ratings.

This research makes several significant contributions. First, it fills the gaps in knowledge regarding service satisfaction in the healthcare industry from caregivers’ perspectives. Second, it identifies the role of empathy in affecting caregivers’ satisfaction and proposes moderators such as the physician–patient communication style to alter the impact of empathy. Finally, this study integrates self-reports and neural methods for empathy recognition, informing the valuable information of neural indicators for health service research.

## Theoretical framework

### Caregivers’ satisfaction with healthcare services: The role of empathy

Traditionally, healthcare service satisfaction has focused on quality-related terms based on patients’ perspectives (Miglietta et al., 2018). In addition to service quality, empathy influences caregivers’ satisfaction, as caregivers do not experience the service themselves but evaluate it through observation.

Empathy denotes the capacity of an observer to feel and understand the emotions, motivations, and behaviors of another human being (de Vignemont and Singer, 2006; Bernhardt and Singer, 2012; Singer and Tusche, 2014). It involves a process of emotional resonance with the other, a state in which the observer experiences and shares the other’s psychological state or feelings. Empathy is beneficial in many contexts (Morelli et al., 2017). In a clinical setting, however, seeing a loved one undergoing a distressing experience might lead the caregivers to experience empathy through which they share the pain and suffering with the patients (De Laurentis et al., 2019; Hua et al., 2021), thereby generating personal distress, anxiety, or unease (Eisenberg and Eggum, 2009; Ghassemi et al., 2020).

Service satisfaction is considered to contain an affective dimension (Liljander and Strandvik, 1997; Reinares-Lara et al., 2019). Generally, compared with positive affect, negative affect is significantly related to dissatisfaction (Westbrook, 1987; Krishnan and Olshavsky, 1995; Ladhari, 2007). In line with these ideas, the distress generated by empathy would impair caregiver satisfaction with healthcare services. The first hypothesis we propose is as follows:

**H1:** Empathy toward patients negatively affects caregivers’ satisfaction with healthcare services.

### Physician–patient communication style: Event-based vs. process-based informed consent

Multiple factors can modulate empathic responses (de Vignemont and Singer, 2006; Yalçın and DiPaola, 2020). Such modulation represents an adaptive advantage in human evolution that makes an individual sensitive to different environmental conditions rather than being overwhelmed by emotions (Hein and Singer, 2008). Here, we propose an essential modulator for caregivers’ empathy, physician–patient communication style, to explore whether specific communication methods can alter empathy’s impact on satisfaction.

Today, the physician’s role is not only as an authoritative person or a medical expert but as a good communicator and collaborator with the patient. Informed consent (IC) has, therefore, been prevalent in improving patient autonomy and physician–patient communication (Lidz, 2011; Stoljar, 2011; Morrison and Sigman, 2021). The doctrine of IC signifies that physicians should disclose all information (e.g., the nature, purpose, risks, benefits, alternatives of medical treatment, etc.) to patients when deciding whether to execute a treatment (Lidz et al., 1988; Brink et al., 2012; Reinares-Lara et al., 2019). However, IC often fails to achieve its original goals and becomes an empty ritual (Morrison et al., 2009; Recchia et al., 2013; Porter et al., 2020).

The problem might not be the doctrine itself but how IC is implemented. IC can be implemented in two ways: the event-based and the process-based model (Lidz et al., 1988; Dodaro and Recchia, 2011; Recchia et al., 2013). The event model emphasizes the validity and comprehensiveness of information disclosure to meet legal requirements. The decision is regarded as a discrete act placed in a short period (usually shortly before the treatment conduction). Whether patients could understand the disclosure is not considered, and in most cases, the information can be too complex to be understood by patients. Patients would feel that their participation in decision-making is under-desired and that their personal situations are not fully considered (Lidz et al., 1988; Feld, 2004; Olson et al., 2012). The process model, however, is built on a series of communicative acts where the decision is determined continuously throughout the diagnostic process. This way, patients can fully consider the risks, benefits, and alternatives. Their autonomy is better fulfilled, and physician–patient communication is enhanced (Spatz et al., 2016).

Effective communication between physicians and patients can improve health outcomes (Ha and Longnecker, 2010; Matusitz and Spear, 2014; Riedl and Schüßler, 2017), which might increase caregivers’ perceptions of the treatment’s effectiveness. Lamm et al. (2007) found that empathic brain responses are reduced when participants are convinced that the therapy is adequate rather than ineffective. They exposed participants to identical video clips, but in one group, the patients received effective treatment, whereas patients from the other group did not benefit. The results showed that witnessing another person suffering and knowing that the treatment had been effective would decrease the empathic responses of the observer. Therefore, the process-based IC is supposed to elicit a lower level of caregiver empathy. Caregivers would then be less affected by patients’ suffering, and the negative effect of empathy could be alleviated. We proposed the second hypothesis as follows:

**H2:** The negative effect of empathy on caregiver satisfaction is more substantial in event-based IC than in process-based IC conditions.

## Materials and methods

### Participants

A total of 30 subjects (16 males and 14 females; *M*_*age*_ = 21.70 years; SD_*age*_ = 2.53 years; age range = 19–30 years) participated in this study. All participants were from Zhejiang University, healthy, had normal or corrected-to-normal vision, were free of hearing problems, and reported no history of neurological disorders or mental diseases. All of them reported having the experience of accompanying someone to clinic visits in recent years.

### Stimuli and procedures

All procedures were approved by the ethics committee of Zhejiang University Neuromanagement Laboratory. Research consent forms were obtained before the experiment. Each participant received 40 RMB after the experiment as compensation. The details of the procedures are as follows.

We used video clips as the experimental stimuli to characterize the responses from a caregiver’s (a third-person) perspective. We recruited an actual physician and standardized patients (SP) to participate in a medical visit. All the video clips were shot at a real hospital and lasted about 2 mins In the process-based IC condition, the physician and the patient had an ongoing dialogue about the risks and benefits of treatment alternatives. The patient made their judgments and discussed personal preferences with the physician. In the end, the patient signed the IC. In the event-based condition, the physician reported information and treatment possibilities to the patient, and the patient passively received all the information and signed the IC. There was not much interaction between the physician and the patient.

Disease severity is an essential factor affecting caregivers’ empathy due to its potential link with perceived pain. Past literature suggests that higher perceived pain leads to increased empathy responses (Singer et al., 2004; Spinella, 2005; Lamm et al., 2007). Therefore, we included disease severity as another potential moderator. Each subject watched four clips, arranged as 2 (communication style: process-based IC vs. event-based IC) × 2 (disease severity: severe vs. mild). In the severe disease condition, the patient had lung cancer, while in the mild condition, the patient had the flu. The four video clips were played in a random sequence.

**FIGURE 1:**
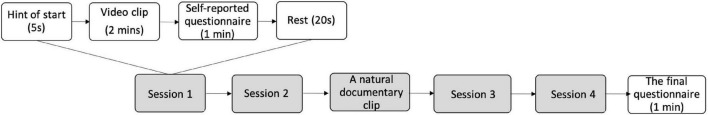
Experiment procedures.

Participants were asked to imagine themselves as caregivers for the patients when watching the videos. They sat comfortably in a moderately lit room with a screen approximately 50 cm in front of their eyes. After each video clip, they were asked to answer a questionnaire about their empathy levels, emotional states, and service satisfaction. When the first two clips finished, a nature documentary clip was played to calm them down to a neutral mood and avoid emotional exhaustion. After finishing all the clips, they filled out another demographic information questionnaire. Their brain activities when watching the videos were recorded *via* the EEG device. The procedures of the experiment are summarized in Figure 1.

### Self-reported measures

A questionnaire was used to assess participants’ self-reported ratings of subjective empathy responses every time they finished watching one video clip. The questions are listed as follows (the answers were assessed on a seven-point Likert scale, 0 = not at all, 7 = completely):

*Experienced empathy* (adapted from Shen, 2010): (1) I can feel the patients’ emotions when watching the videos. (2) I can understand what the patient was going through in the situation. (3) When watching the video, I was fully absorbed (α = 0.89, Barlett’s χ^2^ = 203.26, *p* = 0.000).

*Service satisfaction* (adapted from Friedman and Churchill, 1987): (1) How willing are you to recommend this hospital to your friends and family? (2) Overall, how satisfied are you with the service interaction depicted in the video? (α = 0.91).

*Service quality*: How much do you agree that the patient got good service?

*Emotional state:* What is your current emotional state now? (1 = totally negative, 7 = totally positive).

### Electroencephalography measures

Recent EEG studies have associated empathy with the suppression of “mu rhythms” (Pineda, 2005; Perry et al., 2010a; Peled-Avron et al., 2018). Mu rhythms are desynchronized, and their power decreases when motor activity is engaged and also when actions executed by others are observed (Cochin et al., 1999; Muthukumaraswamy et al., 2004). Such visual–motor coupling suggested by this pattern can reflect a resonance system, which may constitute the biological basis for the simulation theory (Pineda, 2005). Several studies have linked mu suppression to higher social information processing, such as the theory of mind (Perry et al., 2010b) and empathy (Cheng et al., 2008a,b). For example, Perry et al. (2010a) found that mu suppression is modulated both by observation of a situation that is potentially painful for the observer and by empathy for the pain of another person. Mu oscillations are best captured over frontoparietal networks by central electrodes C3, C4, and Cz (Perry et al., 2010a; Horan et al., 2014). Here, we averaged C3 and C4 to capture mu rhythm as a neural indicator for caregiver empathy towards the patient.

### Electroencephalography preprocessing

We used an eight-channel portable EEG device (neurotech, BYS-1524W-S50) for EEG data acquisition. Continuous EEG activity was recorded at a sampling rate of 500 Hz with a notch filter of 50 Hz. EEG signals were measured from Fp1, Fp2, T3, T4, C3, C4, O1, and O2 according to the 10–20 international system of 64 channels and referenced to the left ear mastoid. Artifacts such as eyeblinks, horizontal eye movements, and muscle activities were removed using an independent component analysis (ICA) algorithm, as implemented in the EEGLAB toolbox in MATLAB_R2016b. The preprocessed and artifact-free data were then submitted to a fast Fourier transform by MATLAB_R2016b to compute mu (8–12 Hz) frequency bands.

We averaged the resulting spectral EEG data per video clip for all participants individually. The filtered signals were squared to obtain signals proportional to the power of the EEG frequency bands. Then, each band was log-transformed (Pfurtscheller, 1992; Allen et al., 2004), as untransformed power values were positively skewed.

## Results

### Mean differences among main variables

We performed the following statistical analyses *via* R (version 3.6.2). Mean-level differences between process-based and event-based IC in variables of interest were examined (see Table 1). Repeated measures ANOVA revealed significant differences between the two conditions in mu rhythm, where process-based IC was related to a lower level of empathy response than the event-based condition [△*M* ± SD = 2.698 ± 4.324, *F*(1,29) = 19.863, *p* < 0.001]. No significant difference was found in self-reported empathy between the two conditions [△*M* ± SD = 0.411 ± 1.899, *F*(1,29) = 2.621, *p* = 0.116]. These results indicate that EEG is more sensitive as an empathy indicator, reflecting subjects’ mental states than self-reported measures. Moreover, the satisfaction of process-based IC conditions is significantly higher than that of event-based IC [△*M* ± SD = 0.583 ± 1.809, *F*(1,29) = 4.791, *p* = 0.037].

**TABLE 1:** Descriptive statistics of main variables by physician–patient communication style.

Variables	Mu *M* (SD)	Self-reported empathy *M* (SD)	Service satisfaction *M* (SD)	Emotion *M* (SD)	Service quality *M* (SD)
Process-based IC	–70.820 (4.077)	4.149 (1.460)	3.633 (1.228)	3.617 (1.236)	3.617 (1.541)
Event-based IC	–73.518 (4.126)	4.561 (1.460)	3.050 (1.484)	3.267 (1.219)	3.300 (1.430)

#### The effect of empathy

Next, we conducted a set of regression analyses to investigate the effect of empathy. Two regression models were run, corresponding to the two indicators of empathy: self-reported empathy (Model 1) and mu (Model 2). All data (except dummy variables) were standardized *via* the *z*-score method before being stored in the regression models. The model specification is defined as follows:


(1) $$\begin{array}{cc} S\,e\,r\,v\,i\,c\,e\,S\,a\,t\,i\,s\,f\,a\,c\,t\,i\,o\,n_{i,j} & =\alpha_{0}+\alpha_{1}\,E\,m\,p\,a\,t\,h\,y_{i,j}+\alpha_{2}\,P\,r\,o\,c\,e\,s\,s\,\_\,I\,C_{j}\\ & +\alpha_{3}\,E\,m\,p\,a\,t\,h\,y_{i,j}\times P\,r\,o\,c\,e\,s\,s\,\_\,I\,C_{j}+\alpha_{4}\\ & S\,e\,r\,v\,i\,c\,e\,Q\,u\,a\,l\,i\,t\,y_{i,j}+\alpha_{5}\,S\,e\,v\,e\,r\,e\,D\,i\,s\,e\,a\,s\,e_{j}\\ & +S\,u\,b\,j\,e\,c\,t_{i}+\varepsilon_{i,j}, \end{array}$$

where the *Service Satisfaction*_*i*,*j*_, *Empathy*_*i*,*j*_, and *Service Quality*_*i*,*j*_ are Participant i’s ratings for video *j*. P⁢r⁢o⁢c⁢e⁢s⁢sI⁢Cj and *SevereDisease*_*j*_ are dummy variables that take the value of 1 if video *j* depicts a process-IC communication style or a severe disease scenario; otherwise, they take 0 if video *j* is about an event-IC or a mild disease case. We controlled for the subject’s fixed effect with *Subject*_*i*_ to capture all time-invariant subject-specific characteristics, such as baseline empathy ability. In this model, we interacted empathy with process-IC to test the incremental effect of process-IC. The satisfaction from the event-IC group is the same as that at baseline. Therefore, the interaction term can reflect whether the effect of empathy on caregiver satisfaction in the process-based IC condition is different from that in event-based IC.

Table 2 reports the effects of empathy on caregivers’ satisfaction with the healthcare service. The coefficients of self-reported empathy (Model 1, β = –0.381, *p* = 0.002) and mu (Model 2, β = 0.467, *p* = 0.002) were both significant, revealing that empathy was negatively associated with caregivers’ satisfaction. These results support H1. We did not find any significant effect on disease severity.

**TABLE 2:** Standardized coefficients from the two regression models.

Predictors	β	SE	*t*	*p*	95% CI
**Model 1 (adjusted *R*^2^ = 0.381)**					
Self-reported empathy	–0.381	0.120	–3.168	0.002**	[–0.621, –0.142]
Process-IC	0.153	0.145	1.061	0.292	[–0.134, 0.442]
Self-reported empathy × process-IC	0.381	0.164	2.319	0.023*	[0.054, 0.708]
Service quality	0.513	0.099	5.175	0.000***	[0.316, 0.711]
Severe disease	0.049	0.146	0.337	0.737	[–0.241, 0.339]
**Model 2 (adjusted *R*^2^ = 0.397)**					
Mu	0.467	0.146	3.204	0.002**	[0.177, 0.757]
Process-IC	0.070	0.153	0.454	0.651	[–0.236, 0.375]
Mu × process-IC	–0.505	0.163	–3.096	0.003**	[–0.829, –0.180]
Service quality	0.554	0.096	5.769	0.000***	[0.363, 0.744]
Severe disease	0.030	0.145	0.210	0.835	[–0.259, 0.319]

β, standardized coefficients; SE, standardized error.

*p < 0.05, **p < 0.01, ***p < 0.001.

**FIGURE 2:**
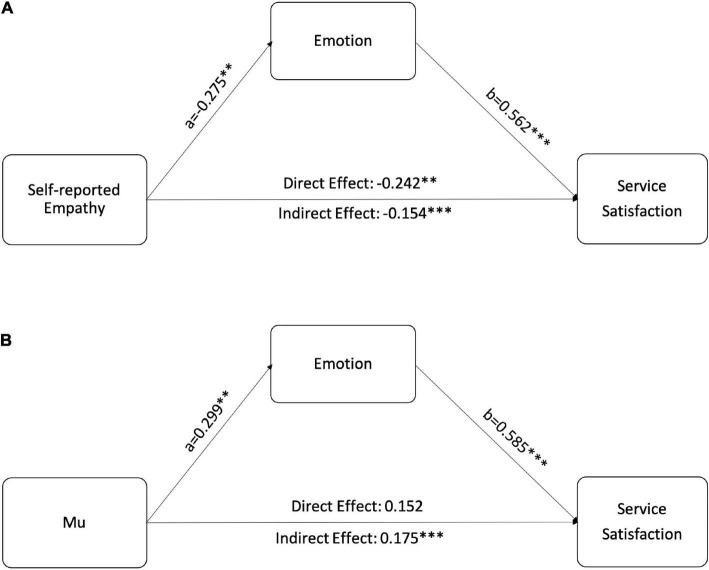
Mediation models of how empathy affects caregivers satisfaction. **(A)** Emotion mediates the relationship between self-reported empathy and satisfaction. **(B)** Emotion mediates the relationship between mu rhythm and satisfaction. **p < 0.01, ***p < 0.001.

To test whether the differences in satisfaction are driven by negative emotions evoked by sharing with patients’ suffering, we ran mediation analyses *via* PROCESS Model 4 with 5,000 bootstrap replications (Hayes, 2017). Disease severity and communication styles were included as control variables. The fixed effect of the subject was also considered. As summarized in Figure 2, both self-reported empathy and mu had a significant relationship with emotions. Emotion mediated the relationship between participants’ empathy responses and satisfaction. These results provide additional evidence for H1 and explain how caregiver empathy decreases their satisfaction with the healthcare service.

#### The moderation effect of physician–patient communication style

Two significant moderation effects were found in Models 1 and 2. The coefficient of self-reported empathy × process-IC was positive and significant (β = 0.381, *p* = 0.023), suggesting that the effect of self-reported empathy on caregivers’ satisfaction in the process-based condition was 0.381 higher than that in the event-based condition. Likewise, the coefficient of mu × process-IC (β = –0.505, *p* = 0.003) suggested that mu’s effect on satisfaction in the process-based condition was 0.505 lower than that in the event-based condition.

**FIGURE 3:**
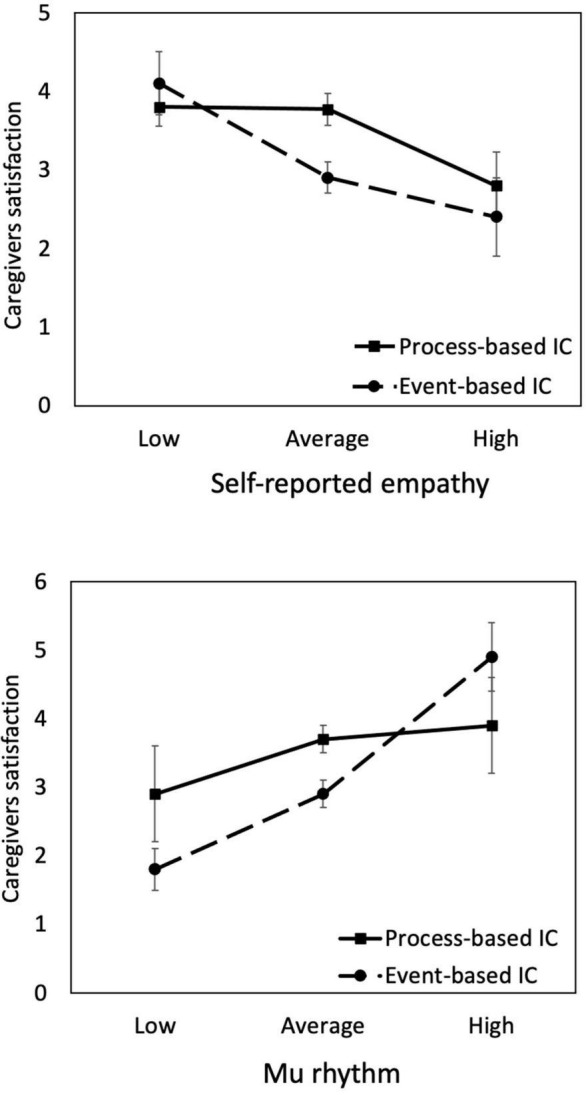
Interactions between empathy (represented by self-reported empathy and mu rhythm) and physician–patient communication style (process-based vs. event-based IC). The negative effect of empathy on caregivers satisfaction is more substantial in the event-IC than process-IC condition. The levels of empathy are plotted at *M* – SD (low), *M* (average), and M + SD (high).

To compare the effect of empathy under the two conditions separately, we conducted four more regression analyses as follows:


(2) $$\begin{array}{cc} S\,e\,r\,v\,i\,c\,e\,S\,a\,t\,i\,s\,f\,a\,c\,t\,i\,o\,n_{i,j} & =\alpha_{0}+\alpha_{1}\,E\,m\,p\,a\,t\,h\,y_{i,j}+\alpha_{2}\,S\,e\,r\,v\,i\,c\,e\\ & Q\,u\,a\,l\,i\,t\,y_{i,j}+\alpha_{3}\,S\,e\,v\,e\,r\,e\,D\,i\,s\,e\,a\,s\,e_{j}\\ & +S\,u\,b\,j\,e\,c\,t_{i}+\varepsilon_{i,j}, \end{array}$$

where all variables are as defined in Equation 1. The results summarized in Table 3 revealed that the coefficients of self-reported empathy (β = –0.370, *p* = 0.020) and the mu (β = 0.544, *p* = 0.023) were significant in the event-IC condition. However, they became nonsignificant in the process-IC condition. These results revealed that the negative effect of self-reported empathy and mu on satisfaction was particularly noticeable when the physician and patient communicated in an event-based way. In other words, the negative effect of empathic responses could be reduced if communication is conducted in a process-based manner. In summary, these findings provide further evidence for H2. The moderation effects are shown in Figure 3.

**TABLE 3:** Standardized coefficients of regression models comparing the effects of empathy under event-based and process-based IC.

	Event-based IC					Process-based IC				
	β	SE	*t*	*p*	95% CI	β	SE	*t*	*p*	95% CI
	**Model 3 (adjusted *R*^2^ = 0.527)**					**Model 4 (adjusted *R*^2^ = 0.475)**				
Self-reported empathy	–0.370	0.150	–2.471	0.020*	[–0.677, –0.063]	0.034	0.136	0.252	0.803	[–0.244, 0.313]
Service quality	0.375	0.155	2.408	0.023*	[0.055, 0.694]	0.625	0.144	4.348	0.000***	[0.330, 0.920]
Severe disease	0.342	0.205	1.669	0.107	[–0.078, 0.763]	–0.316	0.176	–1.795	0.084	[–0.678,0.045]
	**Model 5 (adjusted *R*^2^ = 0.522)**					**Model 6 (adjusted *R*^2^ = 0.473)**				
Mu	0.544	0.226	2.408	0.023*	[0.081, 1.008]	0.002	0.231	0.010	0.992	[–0.472, 0.476]
Service quality	0.554	0.150	3.707	0.001***	[0.247, 0.861]	0.617	0.154	4.009	0.000***	[0.301, 0.933]
Severe disease	0.243	0.218	1.112	0.276	[–0.205, 0.690]	–0.318	0.177	–1.798	0.083	[–0.680, 0.045]

β, standardized coefficients; SE, standardized error.

*p < 0.05, ***p < 0.001.

#### Relationship between the two indicators: Self-reported empathy and mu rhythm

A correlation analysis suggests that mu and self-reported empathy were significantly associated (β = –0.296, *p* = 0.001). Then, we analyzed another regression model (see Table 4, Model 7), including both mu and self-reported empathy. The coefficients in Model 7 suggested that self-reported empathy (β = –0.264, *p* = 0.042) and mu (β = 0.374, *p* = 0.015) were both significant predictors of caregiver satisfaction when added to the same regression model. Mu’s coefficient was more significant than self-reported empathy, indicating that mu is more potent in reflecting empathy responses than stated measures.

**TABLE 4:** Standardized coefficients of Model 7.

Predictors	β	SE	*t*	*p*	95% CI
**Model 7 (adjusted *R*^2^ = 0.412)**					
Self-reported empathy	–0.264	0.128	–2.064	0.042*	[–0.518, –0.010]
Mu	0.374	0.151	2.473	0.015*	[0.073, 0.674]
Process-IC	0.068	0.151	0.447	0.656	[–0.234, 0.369]
Self-reported empathy × process-IC	0.247	0.174	1.418	0.160	[–0.100, 0.593]
Mu × process-IC	–0.362	0.178	–2.040	0.045*	[–0.716, –0.009]
Service quality	0.523	0.097	5.402	0.000***	[0.330, 0.714]
Severe disease	0.000	0.144	0.002	0.998	[–0.287, 0.287]

β, standardized coefficients; SE, standardized error.

*p < 0.05, ***p < 0.001.

**FIGURE 4:**
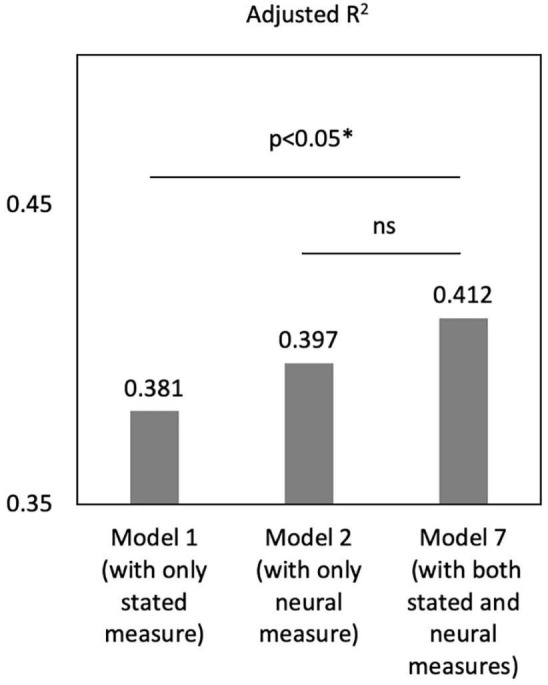
The model fit comparison of model 1, 2 and 7. *p < 0.05.

Notably, the adjusted *R*^2^ of Model 7 is significantly larger than that of Model 1 [Figure 4, *F*(83,85) = 3.28, *p* = 0.043], while there was no difference between Model 2 and 3 [*F*(83,85) = 2.13, *p* = 0.125]. These results suggested that, compared with the model including self-reports only, adding the neural indicator of mu could significantly increase the model fit. However, adding the stated empathy to the model of neural indicator did not change the model fit. Thus, neural signals performed better in predicting caregivers’ satisfaction than self-reported measures, and they two could complement each other to provided added value in predictive power.

## Discussion and conclusion

In the past, we studied service management from a bilateral perspective—the server and the served. Nevertheless, in reality, many services are multilateral. Healthcare service is a typical example. The perception of service quality comes not only from the patient and the doctor but also from the caregiver (Schilling et al., 2002; Turabian et al., 2016). However, a precise mechanism for the third-party bystander’s satisfaction has yet to be identified. We suggest that caregivers’ satisfaction is affected not only by service quality but also by their empathy toward patients, which allows them to understand and respond appropriately to patients’ situations. In this work, we found the “dark side” of empathy: it harms the caregiver’s satisfaction with healthcare services. However, a process-based IC can be an effective way to alleviate such adverse effects. We found evidence through EEG signals and self-reported questionnaires. The details are discussed below.

Our findings suggest that caregivers’ empathy toward patients harms their satisfaction with the healthcare service. This result is reasonable because empathizing with patients can lead caregivers to perceive and resonate with patients’ suffering, distress, anxiety, discomfort, etc. Negative emotions, in turn, evoke lower service satisfaction. Nevertheless, physicians could adopt the process-based IC to alleviate the negative effect of empathy. The process-based IC emphasizes physician–patient mutual involvement in medical consultation, which is likely to result in treatment decisions better reflecting unique individual circumstances and specifically tailored to the patient, and then improve patient satisfaction (Murray et al., 2007; Barry and Edgman-Levitan, 2012; Slade, 2017). According to our work, the process-based IC benefits not only patients’ but also caregivers’ satisfaction.

Through caregivers’ eyes, what they observe in a process-based IC is that the physician and the patient communicate to select appropriate treatments, discussing alternative options, outcomes, uncertainties, and the patient’s preferences in all decision-making stages. They would feel that the patient’s autonomy is better fulfilled, and the patient’s personal preferences and conditions are well considered; thus, the appropriate treatment would be conducted and yield better clinical outcomes. Such belief increases caregivers’ perceptions of the effectiveness of the treatment, provides them with more confidence in the treatment outcome, and reduces the impact of emotion on evaluation.

The current research also compared the validity of using self-reports and neural indicators to reflect empathy. Our experiment did not find significant differences in self-reported empathy between process-based and event-based IC, while mu differences were observed. Moreover, the regression model with both mu and self-reported empathy added value in predictive power beyond the model with only one of them. The absolute value of mu’s coefficient was larger than that of the self-reported measure. Therefore, mu rhythm can be more sensitive and powerful at reflecting empathy than traditional self-reports. According to previous research, the neurological basis of empathy is the mirror neuron system (MNS), in which neurons represent others’ actions in one’s mind (Gallese, 2001; Carr et al., 2003; Lamm and Majdandžić, 2015). Many pieces of research have confirmed the validity of mu suppression as a marker of mirror neuron performance (Muthukumaraswamy and Johnson, 2004; Oberman et al., 2005). Moreover, mu suppression has been associated with better performance in tasks that require emotional empathy (Pineda and Hecht, 2009; Hobson and Bishop, 2017). Empathy is a composite concept consisting of emotional empathy and cognitive empathy as primary forms (Zaki and Ochsner, 2012). Therefore, we believe that self-reports can capture participants’ overall, somewhat obscured, feeling of empathy, while mu reflects the specific facet of emotional empathy. We suggest positioning neuro-data as complementary to traditional data. The combination could create significant advances in the service management field. To our knowledge, few researchers have applied EEG-based techniques to study empathy in the healthcare service field, and we hope this research can serve as a starting point.

Our findings are likely to be of high interest to scholars and the general public. For psychologists and service-science researchers, we provide novel insights into empathy’s “dark-side” effect by integrating the caregivers’ perspectives and showing how to mitigate the adverse effect. For neuroscientists, our findings provide practical evidence to support the utilization of mu rhythm as a representation for empathy response and manifest its usefulness compared with traditional measures. Finally, for physicians and other medical practitioners, our study highlights the importance of effective physician–patient interaction in treatment decision-making. IC should involve a process—more than a signature on a standardized form.

Specifically, it is suggested to conduct an interaction where the physician includes the patient in all stages of the decision-making process. A physician should try to make the patients feel that their needs, values, and preferences are fully respected. Patients, in turn, should be educated on their essential role in decision-making and be given effective tools to help them understand their options and the consequences of their decisions. Patients should also receive emotional support to express their values and preferences and be able to ask questions without time pressure or censure from their physicians. Importantly, patients should be offered decision authority when the choices are related to their values (such as the patient’s religious or moral beliefs) or personal preferences (such as the patient’s insistence on some seemingly “not rational” choices). The physician may give suggestions, but both the physician and the patient decide on the final treatment decisions. Facilitating such communication skills has profound significance in improving healthcare service satisfaction.

## Limitations and directions for future research

There are some limitations to the current research. First, the sample size was not large, and more participants should be recruited to establish findings for the general population. Second, we set the study in a positive scene where the treatment was successful, evoking feelings of “satisfaction.” Whether “dissatisfaction” will be amplified by empathy in negative scenes requires further research. Finally, other factors that may affect an empathy response (such as emotion regulation, trait empathy, self vs. other perspectives, etc.) should be considered. Future research could consider these issues to shed light on healthcare service research.

## Data availability statement

The raw data supporting the conclusions of this article will be made available by the authors, without undue reservation.

## Ethics statement

The studies involving human participants were reviewed and approved by the Ethics Committee of Neuromanagement Laboratory at Zhejiang University. The patients/participants provided their written informed consent to participate in this study.

## Author contributions

XW and RW designed the experiment. RW conducted the experiment and collected the data. XW, RW, and FS interpreted and analyzed the data. RW wrote the main manuscript text. LC helped to conduct the experiment and edited the manuscript. All authors reviewed the manuscript.
