# Corrigendum: The effects of empathy by caregivers on healthcare service satisfaction

**DOI:** 10.3389/fpsyg.2022.1101500

**Published:** 2022-12-01

**Authors:** Xiaoyi Wang, Ruining Wang, Feng Sheng, Leyi Chen

**Affiliations:** ^1^School of Management, Zhejiang University, Hangzhou, China; ^2^Faculty of Arts, McGill University, Montreal, QC, Canada

**Keywords:** service satisfaction, physician–patient communication, empathy, patient-centered care, patient autonomy, electroencephalography (EEG)

In the published article, there was an error in the Funding statement. The original article did not include the funding information. The correct Funding statement appears below.

## Funding

This research was supported by Grant No. 72072161 from the National Natural Science Foundation of China and the Fundamental Research Funds for the Central Universities 2022.

The authors apologize for this error and state that this does not change the scientific conclusions of the article in any way. The original article has been updated.

## Publisher's note

All claims expressed in this article are solely those of the authors and do not necessarily represent those of their affiliated organizations, or those of the publisher, the editors and the reviewers. Any product that may be evaluated in this article, or claim that may be made by its manufacturer, is not guaranteed or endorsed by the publisher.

